# Epidemiology of lung cancer in Northern Greece: An 18-year hospital-based cohort study focused on the differences between smokers and non-smokers

**DOI:** 10.18332/tid/118718

**Published:** 2020-03-24

**Authors:** Kalliopi Domvri, Konstantinos Porpodis, Panagiota Zisi, Apostolos Apostolopoulos, Angeliki Cheva, Theodora Papamitsou, Despoina Papakosta, Theodoros Kontakiotis

**Affiliations:** 1 Department of Respiratory Medicine, Aristotle University of Thessaloniki, George Papanikolaou Hospital, Thessaloniki, Greece; 2 Laboratory of Pathology, Medical School, Aristotle University of Thessaloniki, Thessaloniki, Greece; 3 Laboratory of Histology-Embryology, Aristotle University of Thessaloniki, Thessaloniki, Greece

**Keywords:** lung cancer, epidemiology, smoking, bronchoscopy, histology

## Abstract

**INTRODUCTION:**

Lung cancer remains a leading cause of cancer incidence, yet, in Greece, country-level registry-based data are limited. We have thus investigated the epidemiology of lung cancer and its trends in the George Papanikolaou Hospital, Northern Greece over 18 years (2000–2018).

**METHODS:**

We analyzed all the cases reported in the Bronchoscopy Unit of the Hospital for the period 2000–2018. In total, 15131 subjects (12300 males and 2831 females) that presented with a mass in the imaging, were submitted to bronchoscopy. Characteristics of patients such as age, sex, smoking history and occupation were collected. Statistical analysis was performed with SPSS 21.0 software package.

**RESULTS:**

Among all subjects, a total of 5628 (37.2%; mean age: 65.85 ± 9.6 years) cases of primary lung cancer were identified with a male to female ratio of 2:1 (41.1% to 20.4%) (p<0.001). Squamous cell lung cancer was the most common type of lung cancer identified in this population (44%) with a higher proportion in males compared to females (p<0.001). Furthermore, adenocarcinoma was mostly observed in female non-smokers compared to males (p<0.001). The majority of lung cancer cases were identified in patients occupied with agriculture and livestock breeding (41.1%). The mean age at lung cancer diagnosis was 66.13 ± 9.19 years for the whole study population. Lung cancer cases observed with a higher mean of 43.93 ± 10.84 years of smoking compared to cancer-free patients with 39.64 ± 13.23 years of smoking (p<0.001).

**CONCLUSIONS:**

Apart from smoking, demographic characteristics including age, sex and occupation appear to have an impact on lung cancer development in this population. Smoking history alone could not predict the development of lung cancer in the studied northern Greek population.

## INTRODUCTION

Lung cancer has been transformed from a rare disease to a leading cause of cancer incidence and mortality worldwide^1^. Industrialization, urbanization and environmental pollution around the world has contributed to the complexity of the etiologic factors of lung cancer. Epidemiological studies try to shed light on the characteristics of lung cancer and its relative risk factors, and play an important role in the tertiary prevention of lung cancer and in exploring new ways of diagnosis and treatment.

Lung cancer is the most commonly diagnosed cancer type in the world with 2.1 million new cases, representing 11.6% of all diagnosed cancer cases and a high mortality accounting for 1.8 million deaths (18.4%) in 2018. More specifically, a total of 1.37 million and 0.73 million new lung cancer cases were reported in men and women, respectively^2^. Lung cancer is broadly divided into two categories according to its histological characteristics: small cell lung cancer (SCLC, about 15% of all lung cancers) and non-small cell lung cancer (NSCLC, about 85%). The latter comprises several histological subtypes, mainly squamous cell carcinoma, adenocarcinoma and large-cell lung cancer; adenocarcinoma has been the most common subtype of NSCLC (about 40%)^3^.

Many etiological factors of lung cancer have been identified including: active cigarette smoking; exposure to secondhand cigarette smoke (passive smoking); pipe and cigar smoking; occupational exposure to agents such as asbestos, nickel, chromium, and arsenic; exposure to radiation, including radon gas in homes and mines; and exposure to indoor and outdoor air pollution^4^. However, tobacco smoking is the most well-established causative factor of all types of lung cancer and accounts for about 85% of all lung cancers in current or former smokers^5,6^. Nevertheless, only approximately 10% of smokers develop lung cancer, with the disease occurring also in the absence of exposure to cigarette smoke^6,7^.

Concerning the link between smoking and lung cancer development, previous studies have demonstrated that among individuals with non-small cell lung cancer, those with a history of smoking had a poorer survival than never smokers^6^. Furthermore, smoking is most strongly related to squamous cell carcinoma and small cell lung cancer, while adenocarcinoma is the predominant type of lung cancer in lifelong never smokers^8^. Also, lung cancer cases among non-smoker females are of increasing concern in developing countries^9^.

Risk factors for lung cancer can be prevented, leading to the reduction of mortality rates, through tobacco control and other population-based preventive strategies^10^. However, global numbers do not show a consistent declining or rising trend in either incidence or mortality rates of lung cancer. Only relatively few specific populations such as in the US, and possibly in the UK, are showing signs of decline among recent birth cohorts^11^. Furthermore, information from European registries, including treatments for lung cancer and their efficacy as well as data on tobacco consumption and mutational profiles of tumors, is somewhat scarce.

Until recently, coverage of the population by cancer registries in Greece was limited, with no official unified database for lung cancer and other thoracic tumors. In the present study, we investigated the epidemiology of lung cancer and its trends in Central Macedonia, Northern Greece over almost 18 years, based on smoking history.

### Study design

The present study is a hospital-based observational study of patient enrollment that started in January 2000 and ended in December 2018. All data were from the Bronchoscopy Unit of George Papanikolaou General Hospital, Exochi, Thessaloniki, which is the main tertiary referral center for pulmonary diseases for Northern Greece. The registry was approved by the George Papanikolaou Hospital ethical committee.

## METHODS

Subjects were retrospectively studied from the archives of the above Bronchoscopy Unit. They presented with mass in their radiological examination, and they were all submitted to bronchoscopy in the Bronchoscopy Unit of the Hospital. All patients gave written consent to be submitted to bronchoscopy. Smoking history and occupation were included in patients’ characteristics. Ex-smokers were defined as subjects who quit in the past 12 months and never smokers those who smoked less than 100 cigarettes in their lifetime.

Diagnosis of lung cancer was based on evidence of malignancy on histology and/or cytology of bronchoscopic specimens. On the basis of histology, patients were classified according to the 1999 WHO histological classification of lung cancer^12^. Upon diagnosis of lung cancer, the histological type and the location of tumor (according to imaging findings) were also included in the analysis. All patients were permanent residents in North Greece in urban or semi-urban regions, and data were only available at the municipality level.

### Statistical analysis

Statistical analysis was performed using the SPSS (version-21.0 IBM-SPSS-statistical-software, Armonk, NY, USA). Descriptive statistics were performed and quantitative data were summarized as means with standard deviation (SD), and medians with range (minimum–maximum). To separate parametric from non-parametric variables normality tests using the Shapiro–Wilk test were performed. Qualitative variables were summarized as frequencies in the entire population and percentages. The differences between groups were determined with a two-tailed Student’s t-test or ANOVA for parametric variables, and Mann–Whitney U test or Kruskal–Wallis test for non-parametric variables. Differences were considered statistically significant at p<0.05 or p<0.001.

## RESULTS

**Figure 1:**
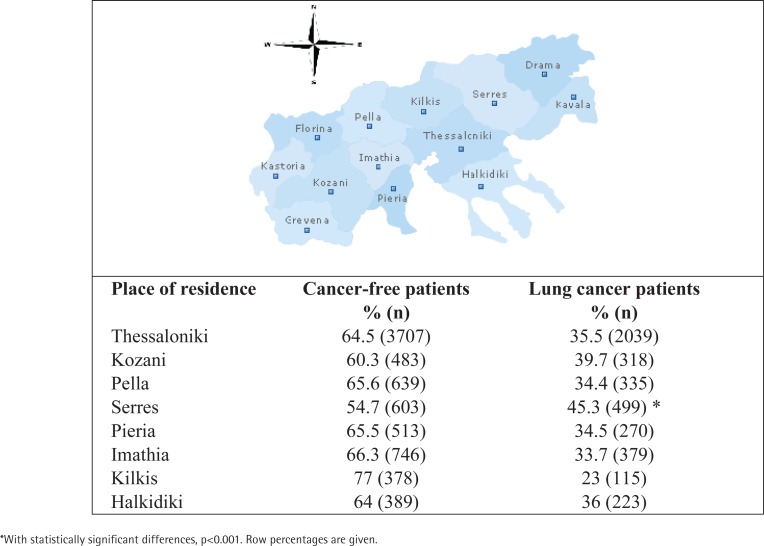
Geographical distribution of the bronchoscopy patient population at the municipality level in Central Macedonia in Northern Greece

Overall, a total of 15131 patients were submitted to bronchoscopy, 12300 male and 2831 women, of which 5636 lung cancer patients were identified in the period 2000–2018. A summary of the patients’ characteristics is given in Tables 1 and 2. Also, the geographical distribution of the whole population at the most frequent municipalities are presented in Figure 1. Most patients were identified from the Serres county (45.3%) (p<0.001). No statistically significant differences were found concerning the place of permanent residence in relation to age, smoking status or occupation.

**Table 1:** Baseline characteristics of the whole population undergoing bronchoscopy, 2000–2018, Thessaloniki, Greece (N=15131)

*Characteristics*	*Mean ± SD or n (%)*
**All patients**	15131 (100)
**Age** (years)	64.02 ± 12
**Sex**	
Male	12300 (81.3)
Female	2831 (18.7)*
**Age at onset of smoking** (years)	19.21 ± 5.79
**Years of smoking** (pack-years)	41.03 ± 12.7 (62)
**Cancer-free patients**	9495 (62.8)
**Smoking status**	
Non-smokers	3066 (32.3) *
Smokers	3532 (37.2) *
Ex-smokers	2896 (30.5) *
**Years of smoking** (pack-years)	39.64 ± 13.23 (60) *
**Age at onset of smoking** (years)	19.41 ± 6 *
**Lung cancer patients**	5636 (37.2)
**Smoking status**	
Non-smokers	639 (11.3) *
Smokers	2852 (50.6) *
Ex-smokers	2147 (38.1) *
**Years of smoking** (pack-years)	43.93 ± 10.84 (65) *
**Age at onset of smoking** (years)	18.93 ± 5.5 *

* With statistically significant differences, p<0.001.

**Table 2:** Characteristics of lung cancer patients undergoing bronchoscopy, 2000–2018, Thessaloniki, Greece (N=5636)

*Characteristics*	*n (%)*
**Age at diagnosis** (years)	
Mean ± SD	65.85 ± 9.6
Median (range)	67 (18–94)
**Sex**	
Male	5059 (90)
Female	577 (10)
**Male smoking status**	
Non-smokers	318 (6.3) *
Smokers	2716 (53.7) *
Ex-smokers	2053 (40.6) *
**Female smoking status**	
Non-smokers	299 (51.8) *
Smokers	119 (20.6) *
Ex-smokers	101 (17.6) *
**Histological type of lung cancer**	
Small cell lung cancer	1275 (23)
Non-small cell lung cancer	
Adenocarcinoma	1231 (22)
Squamous cell carcinoma	2484 (44)*
Other	646 (11)

* With statistically significant differences, p<0.001. Percentages given for each characteristic.

### Age

The study included individuals aged 18–95 years with a mean age of 64 ± 12 years. Out of the 5636 lung cancer patients, the highest percentages occurred in the age groups 61–70 (37%) and 71–80 (32.4%) years (p<0.001). Results of age distribution are shown in Table 3. The number of people aged 51–60 (20.3%) is also significant (p<0.001), with the smallest percentages occurring in age groups ≤30, 31–40 and >80 years. The largest proportion of non-smokers and ex-smokers were noted over the age of 60 years.

**Table 3:** Age distribution based on smoking status of lung cancer patients undergoing bronchoscopy, 2000– 2018, Thessaloniki, Greece (N=5636)

*Age (years)*	*Patients n (%)*	*Non-smokers n (%)*	*Smokers n (%)*	*Ex-smokers n (%)*
≤30	13 (0.2)	4 (1.2)	6 (0.1)	3 (0.3)
31–40	48 (0.9)	8 (2.5)	34 (0.8)	6 (0.5)
41–50	333 (5.9)	22 (6.8)	277 (11)	34 (3.1)
51–60	1144 (20.3)*	35 (10.8)	930 (22)	179 (16.3)
61–70	2080 (37)*	93 (28.7)*	1589 (38)*	398 (36.2) *
71–80	1823 (32.3)*	137 (42.3)*	1258 (29.8)*	435 (39.6) *
>80	192 (3.4)	23 (7.1)	126 (3)	43 (3.9)
Total	5636	324	4214	1098

* With statistically significant differences, p<0.001. Column percentages are given.

### Smoking status

Of the 5636 patients, 324 patients (6%) had no history of smoking (non-smokers) at any time in their life compared to 5312 patients (84%) who had smoked at some point (ex-smokers). A larger proportion of male cancer patients were current or ex-smokers (94.3%) compared to female cancer patients (48.2%) (p<0.001). Results of smoking in lung cancer patients are shown in Tables 2, 3 and 5. Smokers comprised 37.2% of the cancer-free patient population. Lung cancer cases observed with a higher mean of 43.93 ± 10.84 years of smoking compared to cancer-free patients with 39.64 ± 13.23 years of smoking (p<0.001).

### Sex

Out of 5636 patients, there were 5059 (90%) males and 577 (10%) females, i.e. corresponding to a male to female ratio of approximately 9:1. A larger proportion of females (66%) were non-smokers compared to only 15% of males who were non-smokers (p<0.001). Results are shown in Table 5 concerning gender and the diagnosis of lung cancer patients, with a male to female ratio of 2:1 (41.1% to 20.4%; p<0.001) (Table 4).

**Table 4:** Male to female ratio in diagnosis of lung cancer among patients undergoing bronchoscopy, 2000–2018, Thessaloniki, Greece

*Characteristics*	*Cancer-free n (%)*	*Lung cancer n (%)*	*Total n*
Male patients	7241 (58.9)	5059 (41.1) *	12300
Female patients	2254 (79.6) *	577 (20.4)	2831
Total	9495 (62.8)	5636 (37.2)	15131

* With statistically significant differences, p<0.001. Row percentages are given.

**Table 5:** Sex and histological type distribution of lung cancer patients undergoing bronchoscopy, 2000–2018, Thessaloniki, Greece

*Smoking status*	*Small cell lung cancer, n (%)*		*Adenocarcinoma, n (%)*		*Squamous cell carcinoma, n (%)*	
	*Male*	*Female*	*Male*	*Female*	*Male*	*Female*
Non-smokers	23 (3.9)	9 (15)	53 (11.5)	94 (70.1) *	56 (4.4)	22 (34.9)
Smokers	354 (60.5) *	33 (55) *	225 (48.9)	27 (20.2)	657 (52.2)	28 (44.5)
Ex-smokers	209 (35.6)	18 (30)	182 (39.6)	13 (9.7)	546 (43.4)	13 (20.6)
Total	586	60	460	134	1259	63

* With statistically significant differences, p<0.001. Column percentages are given.

### Histology

Small cell lung cancer was diagnosed in 1275 patients (22.6%), while 66% of the patients had NSCLC (p<0.001). Results of histology distribution of lung cancer patients are shown in Table 5. Within NSCLC, the most common histology was squamous cell carcinoma (44%) followed by adenocarcinoma (22%). There was a statistically higher occurrence of adenocarcinoma in non-smoker females compared to male non-smoker patients (p<0.001). Results of gender distribution in histology types are shown in Table 6. Small-cell and squamous carcinoma were more commonly found among men (p<0.001) and smokers, than in non-smokers (p<0.05).

**Table 6:** Gender distribution in histological types of lung cancer among patients undergoing bronchoscopy, 2000–2018, Thessaloniki, Greece (N=5636)

*Histological type*	*Male n (%)*	*Female n (%)*	*Total n (%)*
Small cell lung cancer	1157 (22.9)	118 (20.4)	1275 (22.6)
Adenocarcinoma	982 (19.4)	249 (43.2) *	1231 (21.8)
Squamous cell carcinoma	2374 (46.9) *	110 (19.1)	2484 (44.1)
Other histological types	546 (10.8)	100 (17.3)	646 (11.5)
Total	5059	577	5636

* With statistically significant differences, p<0.001. Column percentages are given.

### Location of the tumor

The most frequent location of tumor or mass was found to be the right upper lobe both in cancer and cancer-free patients (p<0.05), followed by left upper lobe for cancer patients and right lower lobe for cancer-free patients. Results of mass/lesion location for the whole population are given in Supplementary Table 1.

### Occupation

Concerning the occupation of the participants, of the 5636 cancer patients, the highest frequency was observed in farmers and livestock breeders (26.2%) (p<0.001). Results are given in Supplementary Table 2. The next largest group consisted of craftsmen (28.8%), followed by freelance workers (14.1%) and private employees (11.3%). In cancer-free patients, craftsmen were the largest group (26%), followed by farmers and livestock breeders (21.2%).

## DISCUSSION

In the present study, we investigated the epidemiological trends of lung cancer in Northern Greece. The most important findings of our study were the identification of tobacco smoking, age, sex and occupation as significant risk factors of lung cancer. Furthermore, male and female smokers, as well as female never smokers, showed increased lung cancer incidences in specific histological types.

The causal relationship between lung cancer and smoking was well established in the 1950s^13^, and many studies since then have recognized the impact of tobacco smoking on lung cancer^14–17^. Furthermore, other studies have also suggested that smoking is an important independent predictor of short-term lung cancer survival^16^.

In the present study, smoking has been identified as an important risk factor for lung cancer development. Similarly, in a population-based cancer registry in Crete, tobacco smoking was found to have a significant impact on lung cancer incidence and patients’ survival, especially in male smokers^14^. In our study, squamous cell carcinoma was the most common type of lung cancer identified in the Greek population with a higher proportion of male smokers, whereas adenocarcinoma was more common in females. Squamous cell carcinoma, rather than adenocarcinoma, has been traditionally thought to be related to smoking. Similar results have been reported in an earlier analysis of data beginning almost four decades ago from the same Hospital, focusing in two-decade differences in another target population (the region of Thrace included)^18^. In contrast, in other populations, adenocarcinoma has been found to be the most common type^19,20^. It is a fact that prior to the 1970s, squamous-cell carcinoma was the most common histological type of NSCLC, however, since about 1975, in the majority of populations a pathologic shift has occurred, reporting adenocarcinoma as the predominant histological subtype of NSCLC^4,21^.

Furthermore, despite the fact that the majority of cases corresponded to males, we found a significantly higher proportion of female non-smokers among lung cancer patients, especially in adenocarcinoma type, consistent with the upward global trend observed in recent years^22^. Specifically, findings in prior studies from China^23^, Japan^24^ and Northern India^20^ reported respectively that 65%, 70% and 94% of the non-smoker lung cancer patients were female. However, in the US, only 9–13% of female lung cancer patients were never smokers^25,26^. There is indeed a geographical variation that is probably due to ethnic and cultural differences, a fact important to consider when evaluating the higher proportion of EGFR mutation in the Asian population^27^. Concerning the sex-based differences, some studies have implied that sex-hormones are involved in the development of lung cancer in never smokers, but data on these findings are inconsistent^17^. More specifically, estrogen β-receptor has been found to be more common in female never smokers with lung cancer. Also, estrogens have been considered an important factor that contributes to lung carcinogenesis, lung cancer growth, metastasis, and affects prognosis^28^. Our results concerning small cell lung carcinoma showed that it was more common in male and female smokers, similar to previous studies^4^.

In our study, the right upper lobe was the most frequent mass location for both cancer and cancer-free patients. Similar results were found in an earlier analysis of bronchoscopic data in Northern Greece^18^, as well as in the Greek REASON observational Registry Study on a small number of patients^29^ concerning the location of the malignancy. In our study, the most common tumor sites were found to be the upper lobes; a plausible explanation could be that the upper lobes are more affected by tobacco smoking^30^. However, according to other studies, concerning tumor location within the lung, smoking has been reported not to predict patients’ survival^31^. Also, among our main findings was that age proved to be another significant risk factor for lung cancer development, as larger proportions of lung cancer incidence were observed among patients aged ≥50 years, in accordance with the literature^32,33^. In addition, as the vast majority of patients included in the present study were permanent residents of urban or semi-urban municipalities, occupational or environmental factors might have hidden effects on lung cancer development, as highlighted in other studies^14,34,35^. Furthermore, it has been reported that different smoking habits between rural and urban regions result in geographical inequalities in lung cancer incidence distribution^34^.

In our study, the most frequent occupations observed in lung cancer patients were farmers, livestock breeders, and craftsmen. Indeed, farming is one of the most common occupations in Greece, a finding observed also in cancer-free patients. Several studies have already suggested the association of pesticides/greenhouse farming and the development of chronic diseases^36^. However, additional studies are needed to demonstrate any association between smoking and other risk factors (such as environmental exposures) and lung cancer development in Greece.

### Limitations

There are some limitations to our study, such as its retrospective study design, though such long-term prospective studies on this subject are too challenging to be performed. Another limitation is that we missed a small percentage of lung cancers diagnosed by surgical lung biopsy or interventional radiology methods, applied in everyday practice in the last 5 years. Although in our study the impact of tobacco smoking on lung cancer development is evident, in accordance with previous studies, a limitation is the lack of detailed data of other potential risk factors such as behavioral, occupational and environmental that might have led to an overestimation of the smoking impact. The investigation of the exposure to any potential carcinogenic compounds, related to the exact place of residence, for each patient was not possible. Although our study design was a hospital-based study with a descriptive approach, this is the first study in Northern Greece that tries to incorporate data for the last 20 years on the epidemiology of lung cancer incidence, focusing on smoking habits and patient occupations.

## CONCLUSIONS

Greece is a country with a high prevalence of smoking, but a national cancer registry has only recently been initiated and data on lung cancer are scarce. Although registry data are lacking at the national Greek level, there still exists a regional population-based cancer registry – the Cancer Registry of Crete. This has been active since 1992, reporting data for more than two decades.

In our study, apart from smoking, demographic characteristics, including age, sex and occupation appear to have an impact on lung cancer development. Smoking history alone could not predict the development of lung cancer in the northern Greek population.

Also, it is crucial to obtain information on lung cancer patients registry-based at country level that might allow further and specific comparison of lung cancer incidence between different regions. Future studies should focus on the identification of non-smoking related risk factors in order to shed light on the additional effects on the increased lung cancer burden, as never smoker patients are considered to have a biologically unique type of lung cancer. Additionally, in future registries, information on stage, as well as on genetic mutations, would be valuable for further comparisons and conclusions.
