# Erratum: Epidemiology of lung cancer in Northern Greece: An 18-year hospital-based cohort study focused on the differences between smokers and non-smokers

**DOI:** 10.18332/tid/120445

**Published:** 2020-04-13

**Authors:** European Publishing Production Team

**Affiliations:** 1 European Publishing, Heraklion, Greece

**Keywords:** lung cancer, epidemiology, smoking, bronchoscopy, histology

**By Kalliopi Domvri, Konstantinos Porpodis, Panagiota Zisi, Apostolos Apostolopoulos, Angeliki Cheva, Theodora Papamitsou, Despoina Papakosta, Theodoros Kontakiotis**

**Tobacco Induced Diseases, Volume 18, Issue March, Pages 1-8**

**Publish date: 24 March 2020**

**DOI:** https://doi.org/10.18332/tid/118718

In the original article, in the first page, in the authors list, the superscripts indicating the authors’ affiliations were mistakenly given as follows:

Kalliopi Domvri^1^,^2^, Konstantinos Porpodis^1^,^2^, Panagiota Zisi^1^,^2^, Apostolos Apostolopoulos^1^,^2^, Angeliki Cheva^3^, Theodora Papamitsou^4^, Despoina Papakosta^1^,^2^, Theodoros Kontakiotis^1^,^2^

The superscripts in the original manuscript are now corrected:

Kalliopi Domvri^1^, Konstantinos Porpodis^1^, Panagiota Zisi^1^, Apostolos Apostolopoulos^1^, Angeliki Cheva^2^, Theodora Papamitsou^3^, Despoina Papakosta^1^, Theodoros Kontakiotis^1^

^1^Department of Respiratory Medicine, Aristotle University of Thessaloniki, George Papanikolaou Hospital, Thessaloniki, Greece

^2^Laboratory of Pathology, Medical School, Aristotle University of Thessaloniki, Thessaloniki, Greece

^3^Laboratory of Histology-Embryology, Aristotle University of Thessaloniki, Thessaloniki, Greece

