# Supplementary material for: Epidemiology of lung cancer in Northern Greece: An 18-year hospital-based cohort study focused on the differences between smokers and non-smokers
Source: Tob Induc Dis. 2020 Mar 24;18:22. doi: 10.18332/tid/118718 (PMC7132575; doi:10.18332/tid/118718)
Supplement: Supplementary file 1 [file TID-18-22-s1.pdf]

Supplementary Table 1. Results of mass lesion location in patients undergoing bronchoscopy, 2000–2018, Thessaloniki, Greece

**Location of mass**	**Lung cancer patients** n (%)	**Cancer-free patients** n (%)
Left lower lobe	508 (9.5)	926 (12.7)
Right lower lobe	504 (9.4)	1196 (16.5)
Left upper lobe	1035 (19.4)	1043 (14.4)
Right upper lobe	1323 (24.8) *	1672 (23) *
Middle lobe	293 (5.5)	890 (12.3)
Mediastinum	354 (6.6)	144 (2)
Left main lobe	401 (7.5)	126 (1.7)
Right main lobe	252 (4.7)	122 (1.7)
Lingula	171 (3.2)	373 (5.1)
Carina	179 (3.4)	131 (1.8)
Larynx - Vocal cords – Epiglottis - Oropharynx	96 (1.8)	111 (1.5)
Trachea	127 (2.4)	121 (1.7)
Other locations	98 (1.9)	408 (5.6)
Total	5341 (100)	7263 (100)

*With statistically significant differences, $p<0.05$.

Supplementary Table 2. Distribution of occupations in patients undergoing bronchoscopy, 2000–2018, Thessaloniki, Greece

**Occupations**	**Lung cancer patients**<br>n (%)	**Cancer-free patients**<br>n (%)
Farmers - Breeders	1082 (26) *	1549 (21.2) *
Craftsmen - Workmen	1195 (28.8) *	1910 (26.2) *
Unemployed - Students	47 (1.1)	161 (2.2)
Private employees	472 (11.4)	817 (11.3)
State employees	301 (7.3)	548 (7.5)
Freelancers	586 (14)	1005 (13.8)
Medical staff - Pharmacists	60 (1.4)	131 (1.8)
Housekeeping	207 (5)	802 (11)
Pensioners	211 (5)	372 (5)
Total	4161 (100)	7295 (100)

*With statistically significant differences, $p<0.001$.

© 2020 Domvri K. et al.
